# Exploring the influencing factors of initial seizure time during modified electroconvulsive therapy in patients under 30 years old with mental disorders

**DOI:** 10.3389/fpsyt.2026.1824249

**Published:** 2026-07-08

**Authors:** Wanwen Li, Mingjian Cai, Xiaoli Sun, Huali Huang, Chao Ding, Shuang Fu, Changjiang Zhu

**Affiliations:** 1 Department of Psychiatry, Affiliated Mental Health Center & Hangzhou Seventh People’s Hospital, Zhejiang University School of Medicine, Hangzhou, China; 2 Department of Information, Affiliated Mental Health Center & Hangzhou Seventh People’s Hospital, Zhejiang University School of Medicine, Hangzhou, China

**Keywords:** age, energy percentage, mental disorders, modified electroconvulsive therapy, random forest classification, seizure time

## Abstract

**Objective:**

To investigate the factors influencing the initial seizure duration during modified electroconvulsive therapy (MECT) in patients under 30 years old with mental disorders and propose optimized initial treatment parameters for this population.

**Methods:**

A total of 287 patients were included. Six features—preset energy percentage, etomidate dose, gender, age, antiepileptic drug usage, and disease type (depression, bipolar disorder, schizophrenia)—were used to build a classification model predicting whether seizure duration was ideal (15–75 seconds) or non-ideal. Data preprocessing included one-hot encoding for categorical variables and Z-score normalization for continuous variables. Synthetic Minority Over-sampling Technique (SMOTE) was applied to balance the dataset. A random forest classification model with five-fold cross-validation was used to analyze feature importance.

**Results:**

The model achieved average precision of 76.9%, recall of 75.1%, and F1-score of 74.4%. Age (importance coefficient: 0.338), energy percentage (0.252), and etomidate dose (0.208) were the core influencing factors, collectively contributing >79% of explanatory power. Disease type, antiepileptic drug usage, and gender had minimal impacts.

**Conclusion:**

For patients under 30 years old, age, energy percentage, and etomidate dose were the most critical predictors of ideal seizure time during initial MECT, while disease type, antiepileptic drug use, and gender had minimal impact. We recommend setting the initial energy percentage to 1/3–2/5 of the patient’s age, combined with etomidate dose adjustment, to effectively optimize seizure duration.

## 1 Introduction

Modified electroconvulsive therapy (MECT) is a first-line physical intervention for acute phases of depression, bipolar disorder, and schizophrenia due to its rapid onset and significant efficacy (1–4). Studies have demonstrated that ECT is also effective for adolescent patients experiencing depressive episodes (5). However, standardized guidelines for MECT in young patients under 30 years old (including adolescents aged 13-18, transitional youth aged 18-26, and young adults aged 26-30) remain lacking (6). Existing studies indicate that seizure duration is closely associated with therapeutic outcomes and adverse effects (7): 15–75 seconds is considered ideal, while deviations significantly increase risks of cognitive impairment and delirium (52.4%) (8–10). Notably, young patients exhibit lower seizure thresholds, often resulting in prolonged seizure durations (>75 seconds) under traditional energy settings (e.g., 1/2-3/4 of age) (11, 12). Nevertheless, research on optimizing initial parameters for this population remains scarce.

Previous studies in China regarding initial energy dosing for MECT have primarily focused on propofol-based anesthesia protocols, with relatively limited research on dose determination under etomidate anesthesia. Current clinical MECT practice predominantly employs age-based empirical determination of initial energy percentage. However, this empirical approach may pose risks of overstimulation in younger patient populations (13, 14). Preliminary clinical observations from our research team revealed that approximately 70% of patients under 30 years old exhibited seizure durations exceeding the optimal threshold when traditional age-based formulas were used for initial energy determination. Although some scholars propose that adjusting initial doses to 1/3 to 2/5 of the age-based calculation might mitigate treatment risks (15), the biological basis of this dose prediction model, its key influencing factors, and population-specific applicability currently lack systematic clinical validation.

This study constructed a random forest classification model using clinical data from 287 patients under 30 years old to: (1) identify core factors influencing initial MECT seizure duration and (2) propose an optimized initial energy percentage protocol for young patients. The findings provide evidence-based guidance for balancing efficacy and safety in clinical practice.

## 2 Method

### 2.1 Study population

This retrospective cohort study included 287 patients who underwent their first bilateral temporal brief-pulse (BT-BP) MECT at Zhejiang University School of Medicine Affiliated Mental Health Center between January and December 2023. Inclusion criteria: (1) diagnosed with depression, bipolar disorder, or schizophrenia per ICD-11; (2) absence of severe somatic diseases (e.g., uncontrolled cardiovascular conditions) or MECT contraindications; (3) complete treatment records.

Among the participants, 60.3% were female (173/287), with a mean age of 22.37 ± 3.98 years. Disease distribution included unipolar depression (21.9%, 63/287), bipolar disorder (44.3%, 127/287), and schizophrenia (33.8%, 97/287). All patients received intravenous etomidate anesthesia (mean dose: 16.87 ± 2.06 mg). Initial energy percentage was set to 10.18 ± 4.84% (calculated as 1/3-2/5 of age).

### 2.2 Treatment protocol

All treatments used the Thymatron System IV device (Somatics, USA). Procedures included:

1. Preoperative preparation: Fasting ≥6 hours; supine positioning with neck extension.
2. Anesthesia: Intravenous atropine (0.5 mg) followed by etomidate (0.3 mg/kg, adjusted to body weight). Anesthesia depth was confirmed by loss of eyelash reflex and verbal response.
3. Electrical Stimulation Parameters: Bitemporal electrode placement was utilized with a fixed pulse width of 0.5 ms. The energy percentage was dynamically adjusted based on the patient’s age (initial setting: 1/3 to 2/5 of the age value). Static impedance was controlled at <1500 Ω, and an uninterruptible power supply (UPS) was uniformly employed to ensure stable current delivery.
4. Monitoring: Real-time EEG seizure duration (from stimulus termination to seizure wave disappearance), SpO2, heart rate, and blood pressure.
5. Postoperative care: Oxygen supplementation for 30 minutes after spontaneous breathing recovery; treatment frequency: 2–3 sessions weekly.

### 2.3 Data collection and processing

#### 2.3.1 Variables

The following data were extracted from the electronic medical record system:

- Independent variables: Age, gender, disease type, antiepileptic drug usage (yes/no), etomidate dose (mg), preset energy percentage (%).
- Dependent variable: Seizure duration (seconds), categorized into two groups based on literature consensus (14–16): ideal duration (15–75 seconds) and non-ideal duration (<15 seconds or >75 seconds).

#### 2.3.2 Data preprocessing

1. Feature encoding: One-hot encoding for categorical variables (gender, disease type, antiepileptic drug usage).
2. Normalization: Z-score standardization for continuous variables (age, etomidate dose, energy percentage).
3. Sample balancing: SMOTE oversampling applied to address class imbalance (ideal: 67.94% vs. non-ideal: 32.06%), achieving a 1:1 ratio.

#### 2.3.3 Model construction and validation

Random Forest, an ensemble learning algorithm based on decision trees, enhances prediction stability and accuracy by aggregating voting results from multiple decision trees. We developed the Random Forest classification model using Python 3.11 and the Scikit-learn library. The dataset was randomly partitioned into training (229 cases) and test sets (58 cases) at an 8:2 ratio. Model performance was evaluated through five-fold cross-validation, with mean values and standard deviations calculated for precision, recall, and F1-score. Feature importance was quantified using the Gini index-weighted averaging method.

### 2.4 Statistical analysis

Independent t-tests compared continuous variables; chi-square tests compared categorical variables (significance: *p* < 0.05).

## 3 Results

**Figure 1:**
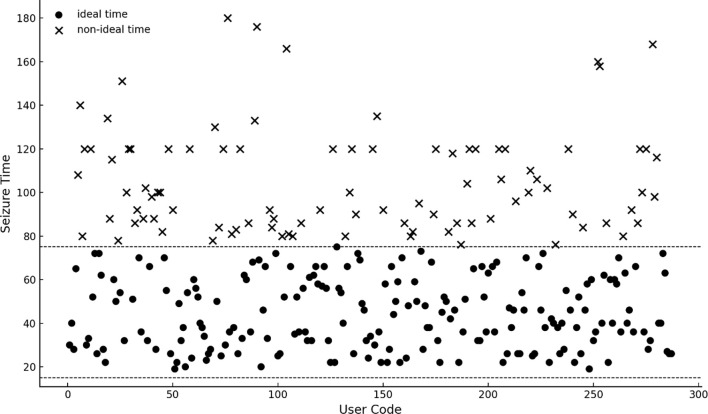
Distribution of seizure duration in 287 patients during initial MECT treatment.

This study included 287 patients undergoing initial Modified Electroconvulsive Therapy (MECT). The median seizure duration was 57 seconds (first quartile: 36 seconds; third quartile: 86 seconds). Based on predefined criteria (ideal range: 15–75 seconds), 195 patients (67.94%) exhibited seizure durations within the ideal range, while 92 patients (32.06%) fell into the non-ideal duration category. The distribution of seizure durations across the cohort is illustrated in **Figure 1**.

For continuous variables, as shown in **Table 1**, the non-ideal group demonstrated significantly higher initial energy percentages compared to the ideal group (10.50 ± 5.61 vs. 9.51 ± 2.36, p = 0.036). However, no statistically significant differences were observed between groups for age (22.52 ± 3.98 years vs. 22.03 ± 4.00 years, p = 0.333) or etomidate dose (16.98 ± 2.17 mg vs. 16.63 ± 1.78 mg, p = 0.151).

**Table 1:** Comparison of continuous variables (*t*-test).

Field	Ideal group	Non-ideal group	T-statistic	P-value
Patient Age	22.52 ± 3.98	22.03 ± 4.00	0.97	0.3329
Etomidate Dose	16.98 ± 2.17	16.63 ± 1.78	1.44	0.1512
Energy Percentage	10.50 ± 5.61	9.51 ± 2.36	2.10	0.0364

For categorical variables, as presented in **Table 2**, no significant intergroup differences were identified for sex (male: 47.56% in the ideal group vs. 33.70% in the non-ideal group; female: 57.44% vs. 66.30%, p = 0.192), disease type (schizophrenia, bipolar disorder, unipolar depression), or antiepileptic medication use (usage rate: 67.18% vs. 66.30%, p = 0.990).

**Table 2:** Comparison of categorical variables (Chi-square test).

Field	Ideal group	Non-ideal group	Chi-square	P-value
Gender			1.70	0.192
- Male	83 (47.56%)	31 (33.70%)		
- Female	112 (57.44%)	61 (66.30%)		
Disease Type			3.175	0.204
- Schizophrenia	69 (35.38%)	28 (30.43%)		
- Bipolar Disorder	89 (45.64%)	38 (41.30%)		
- Unipolar Depression	37 (18.97%)	26 (28.26%)		
Antiepileptic Drug Usage			0.0002	0.990
- Use Drug	131 (67.18%)	61 (66.30%)		
- Non-use Drug	64 (32.82%)	31 (33.70%)		

The five-fold cross-validation results of the Random Forest classification model demonstrated moderate discriminatory power in predicting whether seizure durations were ideal, with mean performance metrics of 76.9% (± 3.2%) precision, 75.1% (± 4.1%) recall, and 74.4% (± 3.8%) F1-score.

**Figure 2:**
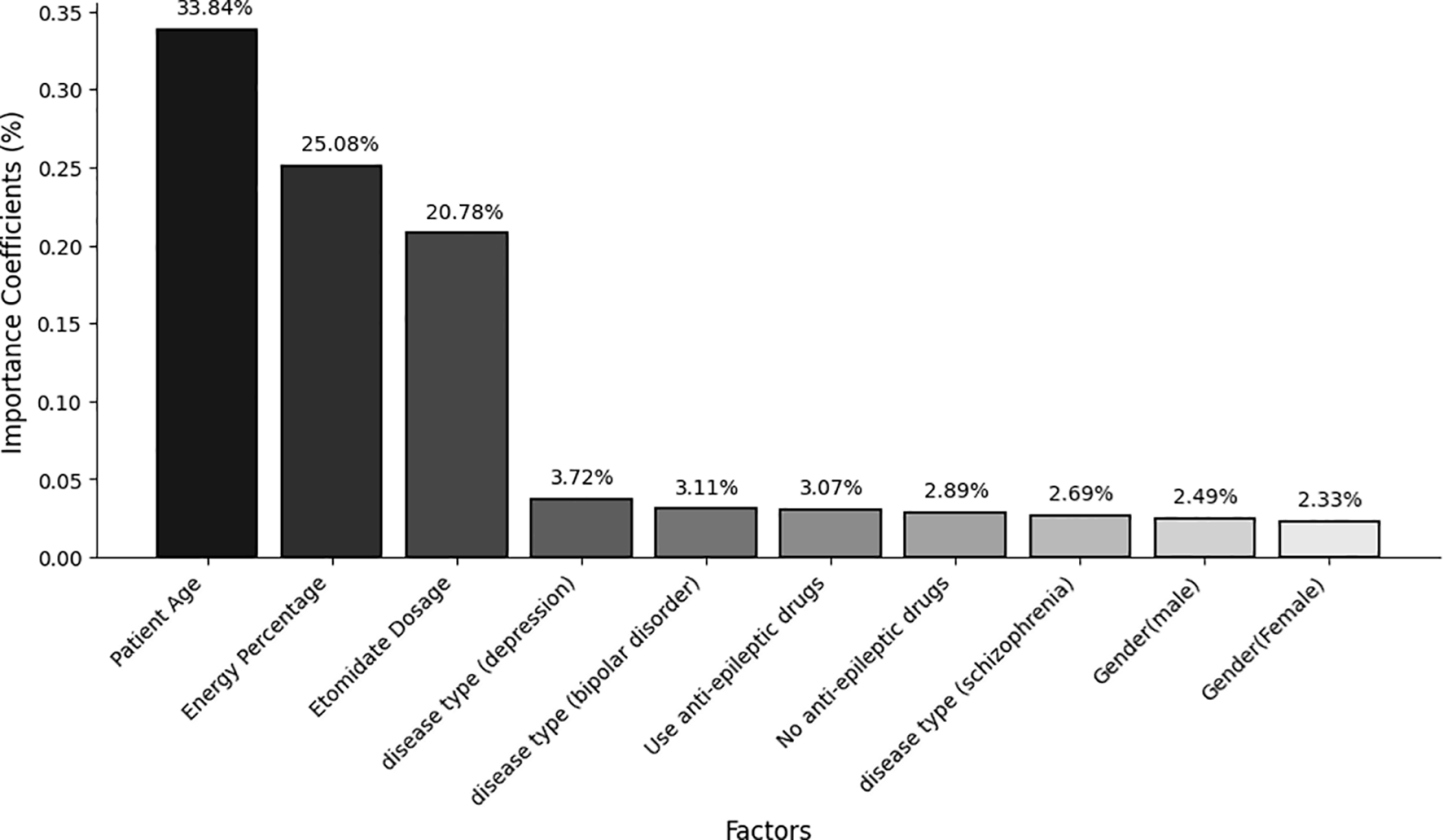
Factor importance coefficients for predicting ideal seizure duration.

**Figure 3:**
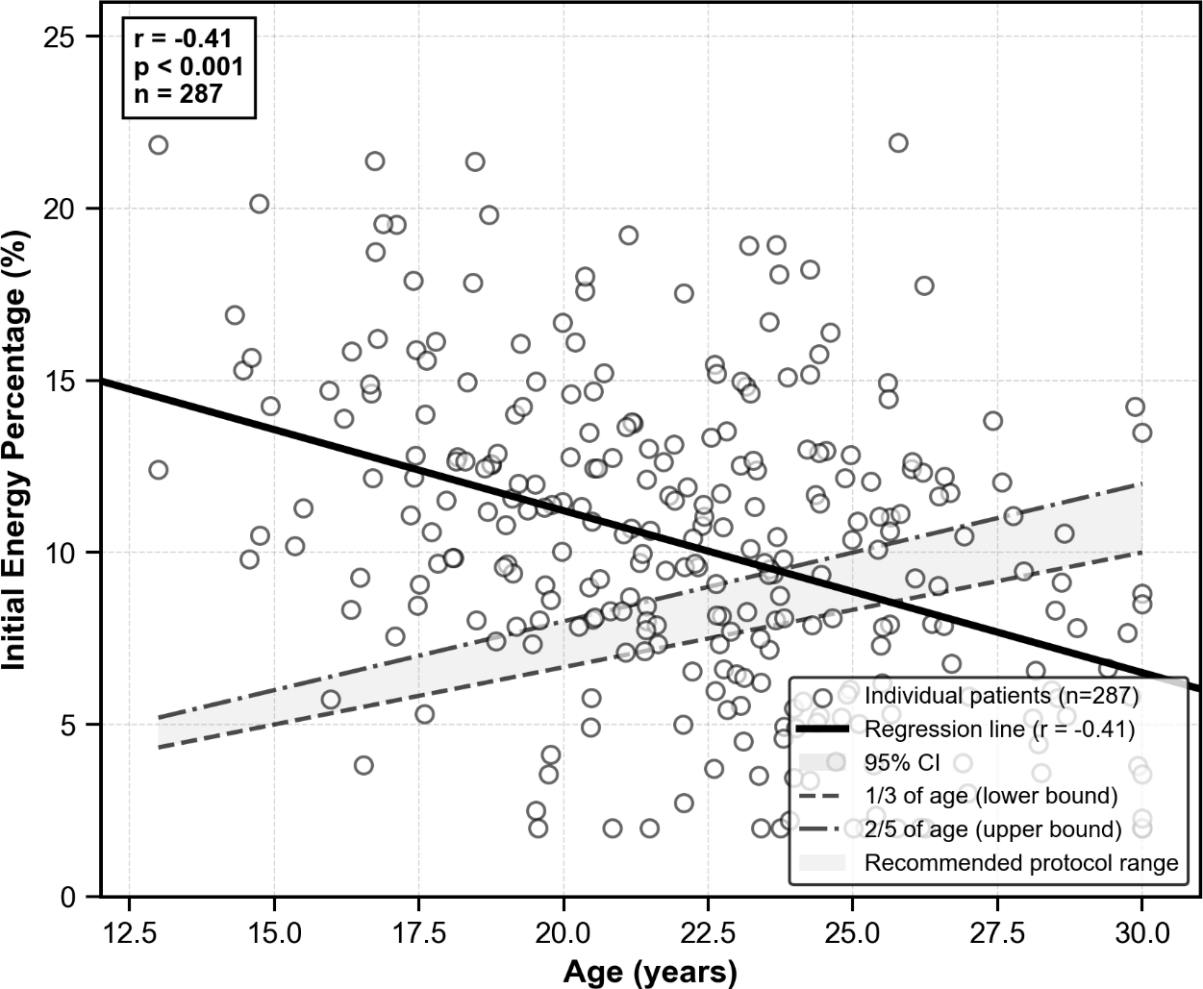
Relationship between patient age and initial energy percentage.

Feature importance ranking based on the Gini index (**Figure 2**) revealed age (importance coefficient: 0.338), initial energy percentage (0.252), and etomidate dose (0.208) as key predictors, with a cumulative contribution of 79.8% to explanatory power. All remaining features exhibited importance values <0.1, including disease type (0.068), sex (0.054), and antiepileptic medication use (0.017). Further analysis identified a significant negative correlation between age and initial energy percentage (**Figure 3**, r = −0.41, p < 0.001), suggesting younger patients require lower energy levels to control seizure duration.

Compared to traditional energy settings (50% of age), the 1/3-2/5 protocol significantly increased ideal seizure duration rates.

## 4 Discussion

This study systematically analyzed factors influencing seizure duration during initial Modified Electroconvulsive Therapy (MECT) in psychiatric patients under 30 years old using a Random Forest model. Results identified age, initial energy percentage, and etomidate dose as core predictors of ideal seizure duration (importance coefficients: 0.338, 0.252, and 0.208, respectively), while disease type, sex, and antiepileptic medication use exhibited minimal influence (all <0.1). These findings diverge significantly from prior studies in adult populations. For instance, Sackeim et al. (12) reported disease type as a critical determinant of seizure threshold, but this association may be attenuated by the biological homogeneity of younger patients in our cohort.

The significant negative correlation between age and initial energy percentage (r = −0.41, p < 0.001) suggests younger patients require lower energy levels to optimize seizure duration. Specifically, adolescents ≤18 years old demonstrated a higher proportion of ideal seizure durations when energy was set at 1/3-2/5 of their age compared to traditional half-age protocols. This aligns with Luccarelli et al. (16), who observed lower seizure thresholds in adolescents than adults, though our study further quantifies age-guided energy calibration. Notably, the non-ideal group exhibited significantly higher energy percentages, indicating overstimulation directly contributes to prolonged seizure durations.

While etomidate’s role in modulating anesthesia depth and seizure activity is well-established (17), its lower importance (0.208) relative to age and energy parameters may reflect younger patients’ faster metabolic rates and interindividual variability in anesthetic sensitivity, destabilizing dose-response relationships. Additionally, the weak positive correlation between etomidate dose and seizure duration (r = 0.12, p = 0.051) implies cautious dose reduction might shorten seizures, though anesthesia safety must be balanced.

This study has several limitations: First, the moderate sample size (n = 287) and single-center design may limit generalizability. Second, the retrospective approach precludes control of confounding factors (e.g., individual baseline EEG variability). Third, long-term cognitive outcomes related to energy settings remain unassessed. Future multicenter prospective studies should integrate neurophysiological biomarkers (e.g., epileptiform wave propagation velocity) to develop dynamic energy adjustment models.

## Data Availability

The raw data supporting the conclusions of this article will be made available by the authors, without undue reservation.
